# The Ecology of a Keystone Seed Disperser, the Ant *Rhytidoponera violacea*

**DOI:** 10.1673/031.010.14118

**Published:** 2010-09-20

**Authors:** Dave Lubertazzi, Maria A. Aliberti Lubertazzi, Neil McCoy, Aaron D. Gove, Jonathan D. Majer, Robert R. Dunn

**Affiliations:** ^1^ Department of Biology, North Carolina State University, Raleigh, NC, USA; ^2^ Department of Plant Science and Entomology, University of Rhode Island, Kingston, Rl, USA; ^3^ Centre for Ecosystem Diversity and Dynamics, Curtin University of Technology, Perth, WA, Australia

**Keywords:** ant-plant interactions, elaiosome, life history, mutualism

## Abstract

*Rhytidoponera violacea* (Forel) (Hymenoptera: Formicidae) is a keystone seed disperser in Kwongan heathl and habitats of southwestern Australia. Like many myrmecochorous ants, little is known about the basic biology of this species. In this study various aspects of the biology of *R. violacea* were examined and the researchers evaluated how these characteristics may influence seed dispersal. *R. violacea* nesting habits (relatively shallow nests), foraging behavior (scramble competitor and lax food selection criteria), and other life history characteristics complement their role as a mutualist that interacts with the seeds of many plant species.

## Introduction

Plant-animal mutualisms typically involve interactions that include numerous partner species (Pellmyr and Thompson 1996; Hoeksema and Bruna 2000; Stanton 2003). A single plant species, for example, may produce seeds that can be dispersed by tens or even hundreds of animal species (Howe and Smallwood 1982; Gaddy 1986; Garrido et al. 2002). However, recent work has shown that even in superficially diffuse seed-dispersal mutualisms, dispersal may depend disproportionately on a few or even just a single species (Boulay et al. 2007; Gove et al. 2007; Zelikova et al. 2008; Ness et al. in press). The biology of such “keystone dispersers” can have ramifying consequences for plant fitness and evolution, but also more generally for the habitats they occur within.

Myrmecochory, the dispersal of seeds by ants, is a common and relatively well studied animal-plant mutualism (Beattie 1985; Bond et al. 1991; Bronstein et al. 2006). It has evolved many times and is geographically widespread (Giladi 2006; Dunn et al. 2007; Lengyel et al. 2009). In this mutualism, ants are enticed to disperse seeds by the presence of elaiosomes, lipid-rich seed appendages that are functionally analogous to fruits (Hughes et al. 1994; Fischer et al. 2008). Elaiosomes are eaten by the ant mutualists after bringing the seeds back to their nest, leaving the seeds unharmed. Seeds are then placed in a refuse dump within the nest or taken out of the nest and discarded. Ants receive nutrients from the interaction, while plants may benefit in two distinct ways. First, a plant's propagules are dispersed away from the parent plant, either in space or time. Second, seeds may be placed in a location that further favors germination and/or establishment (see Beattie 1985, Giladi 2006, and Rico-Gray and Olivera 2007 for reviews of myrmecochory and its potential advantages).

Similar to seed dispersal mutualisms more generally, myrmecochory has typically been viewed as a diffuse mutualism. However, recent work suggests that at least in two of the regions where myrmecochory is common, eastern North America (Ness et al. in press; Zelikova et al. 2008) and southwestern Australia (Gove et al. 2007; McCoy 2008), seed dispersal is dominated by a single genus or species of ant. In both cases, the particular ant species disperses the seeds of tens or, in the case of southwestern Australia, hundreds of plant species. In this context, the life history of these keystone ants becomes important for understanding seed dispersal and the dynamics of local communities.

Perhaps because of their relative ease of study (when compared to, for example, tracking frugivorous birds; Westcott and Devon 2000), many studies have examined the interactions between ants and the seeds they disperse (Berg 1975; Beattie 1985; Bond et al. 1991; Boulay et al. 2007). Much of this work has examined specific aspects of how ants interact with seeds, such as dispersal distances (Gomez and Espadaler 1998; Whitney 2002; Ness et al. 2004), foraging behavior (Hughes et al. 1994; Gorb and Gorb 1999, 2000), and relationships between diaspore morphology and ant workers (Berg 1975; Hughes and Westoby 1992a; Rodgerson 1998; Garrido et al. 2002; Ness et al. 2004). With some important exceptions (e.g. Culver and Beattie 1978; Christian and Stanton 2004; Giladi 2006; Ness et al. in press), the potential relationships among ant life history traits, colony level characteristics, and how these may influence seed dispersal have not yet been investigated as thoroughly. Knowing how a particular ant species will collect elaiosome-bearing seeds and over what distance they may move them, is relatively incomplete information. Nesting chamber depths, the location of refuse middens (including discarded seeds that have been stripped of their elaiosome), and how often nests are abandoned can also influence myrmecochory-related benefits for plants. With little of this type of data available, it remains difficult to gauge how well we truly understand the ecological or evolutionary dynamics of ant-mediated seed dispersal.

This study examined the biology of the keystone ant mutualist *Rhytidoponera violacea* (Forel) (Hymenoptera: Formicidae). In areas where *R. violacea* is found, it appears to be the dominant ant responsible for seed dispersal (Majer 1984; Gove et al. 2007; McCoy 2008). Despite its importance, the basic biology of this species has been largely unexamined. The demography and nesting biology of *R. violacea* were studied to determine the size of their colonies and to examine the physical structure of their nests. Aspects of their foraging behavior were also investigated. Traits found that were salient to seed dispersal and seed fate are discussed in terms of their potential influence on this ant's mutualistic plant partners.

**Figure 1.**
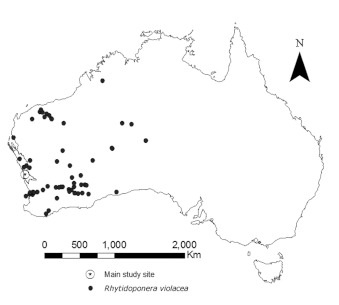
Distribution of *Rhytidoponera violacea*. Collections records are from the Australian National Insect Collection database. The boundary of the Geraldton Sandplains, found along the central western coast, is outlined and the circled star shows the approximate location of Eneabba. High quality figures are available online.

## Materials and Methods

### Study species

Ants of the genus *Rhytidoponera* are distributed throughout Australia and are important seed dispersers in all regions of the continent yet studied (Berg 1975; Majer 1982, 1984; Hughes and Westoby 1992a, 1992b; Gove et al. 2007). A recent revision of the genus (Reichel 2003) shows the species *R. violacea* as having a large range within western Australia (Figure 1). Within its range, *R. violacea* is patchily distributed. At least in the Geraldton Sandplains and perhaps more generally, its abundance is greatest in sites 3 to 15 years after a fire (McCoy 2008). Relatively little is known about the biology of *R. violacea* (Searle 1978), except for what can be inferred from other *Rhytidoponera* species (Haskins and Haskins 1979; Ward 1981a; Pamilo et al. 1985; Thomas 2003).

### Study Area

Research was conducted in the Geraldton Sandplains, a large area situated along the mid-west coast of western Australia. The study area was near the site that first led to the region being recognized as a global biodiversity hotspot (Lamont et al. 1977), a distinction based on the high degree of plant endemism and high species turnover. A large proportion of the plant species in the area produce myrmecochorous seeds.

The Eneabba landscape is a mosaic of natural Kwongan heathland and developed farmland. The vegetation in natural areas varies as a consequence of subtle differences in local topographic position, soil type, and fire history (Hnatiuk and Hopkins 1981). Fire intervals can range from a few to more than 30 years, with the height and density of the vegetation being influenced by the time since the last fire (Westcott 2004; McCoy 2008).

*R. violacea* was studied at two multi-hectare plots located just north of Eneabba and on opposite sides of the Brand Highway (29° 37′ 33″ S, 115° 12′ 59″ E). Both areas contained native Kwongan Heathland, but varied in the time since their last fire. The plot east of the highway (S1) was burned roughly nine months prior to the study. The ground was dominated by open sandy areas with small unburnt islands of vegetation. Many plants in the burnt area were beginning to generate new growth. The second plot (S5) was located west of the highway and was last burned in 2002, five years before sampling. This vegetation covered more than half of the ground surface and in some areas was more than a meter tall.

Foraging metrics between the two sites, as detailed below, were compared using t-tests. Data are reported as means ± SE.

### Diet

A keystone ant species can play a disproportionate role in shaping plant communities by dispersing the seeds of a diverse collection of plant species. *R. violacea* appears to disperse most of the individual seeds of all of the ant-dispersed plant species that have been studied across the Eneabba landscape (Gove et al. 2007). In a pilot study, in which smoke-water was used to stimulate germination of seeds found in 14 *R. violacea* nests, 15 plant species germinated (RR Dunn, personal observation). Control treatments, consisting of soil taken near each colony, resulted in the germination of only four plant species all of which produce wind-dispersed seeds. While these results are consistent with many plant species potentially realizing substantial benefits from seed dispersal by *R. violacea*, the reverse need not be true. Elaiosomes may comprise only a minor portion of the diet of most seed-dispersing ants (Majer 1982; Bono and Heithaus 2002; but see Morales and Heithaus 1998). Thus, an important first step towards assessing the role elaiosomes play in the diet of *R. violacea* is to simply know what their workers collect when they forage.

To determine the composition of the items retrieved, *R. violacea* foragers were sampled as they returned to the nest. During the spring and summer *R. violacea* forage during a morning period, stop foraging during the heat of midday, and forage again in the late afternoon and early evening (JD Majer, unpublished data). Foragers were therefore sampled from 7:00 to 10:00 and from 17:00 to 19:00 in both sites during November 2007.

By watching for ants near the nest entrance, foragers could be observed and captured as they returned to the nest. *R. violacea* is a member of the subfamily Ectatomminae, which cannot store large quantities of liquid in their crop (Eisner 1957). It was assumed that liquid resources were collected at a negligible rate and that workers that returned to the nest without an object in their mandibles were unsuccessful foragers. Workers observed carrying an object were aspirated into a vial. The foraged material was separated from the ant, saved, and the forager returned to the capture location. Foraged items were later examined and assigned to one of the following six categories: 1) insects, live or dead insects and insect parts; 2) pieces of plant material, primarily flower parts and leaf fragments; 3) inert, small clumps of sand, charcoal, etc.; 4) plant seeds; 5) a combination: fragmented parts of two classes, such as an insect part and piece of plant that were stuck together; or, 6) unknown, items that were unclassifiable as either plant or insect material.

A total of 36 nests (18 in each site) were sampled, with each colony being sampled for 30 minutes. Sampling foragers from a nest for this amount of time produced no detectable changes in a colony's foraging dynamics.

### Foraging Distance

Seed-dispersal distances are primarily a function of how far ants forage. The average foraging distance for colonies was found by following randomly encountered ants back to the nest. Individual ants were located by standing in place and scanning any open sandy areas in a roughly circular area (in an approximate radius of 3 m). If no ants were found after a few minutes, a new search was done 10 m from the previous patch. When an ant was located, it was offered a small piece of sweetened oats. These foragers would readily pick up the oats and run back to the nest with this food. The distance from the initial location of the forager to the nest entrance was measured to the nearest 5 cm. Foragers were sampled at both study sites in December 2007.

### Disposal of Seed Proxies

Studies of seed dispersal have generally quantified distances from where a seed is picked up to the nest entrance where a seed is taken. However, seeds can also be thrown out of the nest once their elaiosomes are eaten. *R. violacea* workers had been observed exiting their nests carrying objects that they subsequently dropped (D Lubertazzi, personal observation). A complete understanding of seed dispersal distances, as well as seed fate, has to include knowing how far workers will forage, and also how, and at what distance, objects are discarded away from the nest (Hughes and Westoby 1992b).

To test how far refuse can be carried, 10 pink beads (2.5 mm diameter) were dropped into the nest entrances of marked colonies during the late afternoon. These nests were revisited two afternoons later and the ground around the nest methodically searched for beads. The search included all the area within a 10 m radius of the nest entrance. The distance from each bead to the nest entrance was recorded to the nearest 5 cm. This same bead searching protocol was then repeated the following day. Fourteen nests were sampled in plot S1 and 15 nests sampled in plot S5.

It was assumed that all of the beads not located remained in the nests, and the colonies were not excavated to confirm their fate. It is likely that some beads were removed and not found since our search was thorough, but not exhaustive. The probability of not finding a discarded bead increased with distance from the nest entrance (the search area increases multiplicatively with the square of the radius). In light of these considerations the sampling provided conservative estimates of the average distance beads were carried.

### Nesting Ecology and Demography

Seed dispersal can be influenced by colony demography (e.g., how many workers are in a colony and how many of those individuals forage) and the location where seeds may be abandoned and buried. Whole nests were excavated to determine the size of colonies (number of individuals) and the shape and size of *R. violacea* nests. Twenty-two nests were sampled at S5 and two nests were excavated from S1.

In the sandplains, *R. violacea* usually build their nest chambers under and within root masses of a number of different plant species (e.g., *Daviesia* spp. or species of Restionaceae). Excavating nests necessarily included removing clods of soil, root clumps, and woody roots that were part of the nest's structure. The ants and these materials were collected both by hand and with a plastic grain scoop and placed into the top of a series of stacked sieves. Once a nest was excavated, the coarser material was separated out and the remaining contents (ants, brood, sand, and some detritus) were placed into a plastic wash bin. These were later brought to the field lab and the ants were allowed to move into artificial nests. On the following day the number of workers, pupae, and larvae were tallied for each nest by counting the contents of the artificial nesting chamber and any individuals remaining in the bin.

Two other types of samples were collected to complement the nest excavation data. Plaster castes were made by pouring dental plaster into a colony's nest entrance. The plaster was allowed to harden for a few days and then dug from the ground, cleaned, measured, and photographed. The arrangement of the chambers and the overall size of complete nests could easily be ascertained from these castes. The size and number of nest entrances from 14 nests were also recorded.

## Results

### Diet

A total of 185 successful foragers were sampled from 36 colonies. The average number of successful foragers returning to the nest over a 30-minute period was 5 ± 0.5 (range = 1 - 14, n = 36). While the numbers of unsuccessful foragers that returned to the nest were not systematically recorded (collecting and processing successful foragers took precedence during sampling), there were typically between 10 to 15 return trips to the nest during 30 minutes of sampling, suggesting that approximately 30 – 50% of foraging bouts are successful. No difference was detected in the number of successful foragers observed per nest in the two plots (t_34_ = 1.34, p = 0.19).

**Figure 2.**
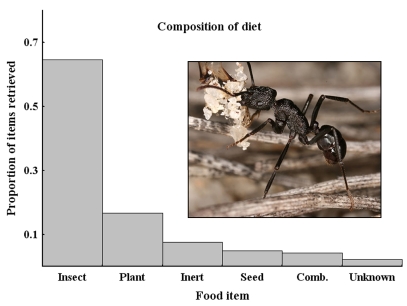
The proportional representation of food items being brought back to the nest by returning *Rhytidoponera violacea* foragers. See methods section for an explanation of the classes. Photo by Benoit Guenard. High quality figures are available online.

The percentage of different categories of foraged items was similar between the two plots hence the data from the two locations were pooled (Figure 2). The majority of items captured (65%) were insects (either whole individuals or insect parts). Plant parts were more commonly collected (17%) than seeds (5%). In a few cases, foragers were observed subduing live insect prey that was then brought back to the nest. In addition, some insects (e.g., non-conspecific ants, small beetles, and termites) were often found to be alive when they were collected from the foragers.

### Foraging Distance

**Figure 3.**
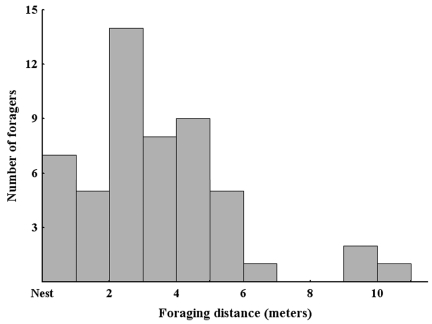
Histogram showing the foraging-distance distribution for 52 randomly encountered *Rhytidoponera violacea* foragers. High quality figures are available online.

The average foraging distance from the nest was 3.5 ± 2.2 m (Figure 3), with a maximum distance of 10.4 m. No difference was observed in the average foraging distance between the two sites (t50 = -1.34, p = 0.18). These distances are similar to those previously observed in the same study region for *R. violacea* carrying seeds back to the nest (Gove et al. 2007; McCoy 2008).

### Disposal of Seed Proxies

More than half of all the beads (150/290) were found outside of the nests. On average these beads were located a quarter of a meter away from the nest entrance (mean = 24 ± 0.04 cm, n = 150 beads). A total of 61 of 140 beads were found from the 14 nests in S1 (mean distance from entrance = 43 ± 9.9 cm, n = 61 beads) and 89 of 150 beads were found from 15 nests in S5 (mean distance from entrance = 11 ± 2.4 cm, n = 89 beads).

### Nest Demography

The average number of workers in a colony was 190 ± 23.5 (range = 47 – 474, n = 22). All of the colonies excavated contained pupae (mean = 83 ± 13.4, range = 2 – 293) and many contained larvae (mean = 22 ± 3.8, range = 0 – 85). Small larvae (< 3 mm) and eggs were either uncommon or entirely absent. A total of seven males were collected from two colonies.

### Nest architecture

**Figure 4.**
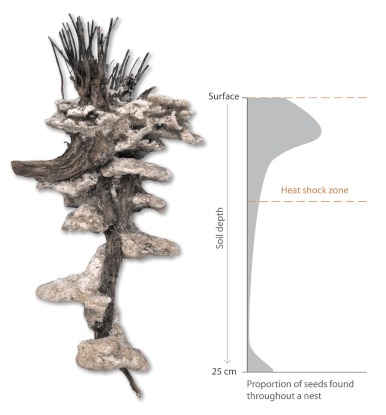
A cast of a typical nest of *Rhytidoponera violacea*. The nest is relatively shallow, with the bottom chamber reaching a depth of 25cm. The proportion of seeds within a nest is shown on the right (n = 6 nests, Dunn et al. 2008). Note that seeds are concentrated near the ground surface and at the deepest nest chambers. The area between the two dashed lines indicates the greatest depth to which a “typical” fire is likely to warm the soil sufficiently to trigger the germination of seeds. High quality figures are available online.

All the excavated nests were located under plants. The nests were either supported in part by a large mass of roots from a plant or, less commonly, incorporated a larger root of a shrub into their structure (Figure 4). The mean (n = 14) size of the nest entrance was 4.0 ± 0.58 cm (longest axis) by 2.1 ± 0.29 cm (perpendicular to widest axis). The number of nest openings varied (9 nests with 1 opening, 3 nests with 2 openings, 1 nest with 3 openings, and 1 nest with 4 openings). When present, multiple nest openings were located within a few centimeters of one another and coalesced into a single chamber or tunnel 1–2 cm below ground. A mound of nest spoil was found around most, but not all, nest entrances. Mounds were typically oval in shape, approximately centered on the nest opening, and often obscured by the stems and shoots of the vegetation of the overlaying plant. The average longest axis width of a mound was 21.0 ± 1 cm, the width perpendicular to the longest axis averaged 15.7 ± 1.4 cm, and the average mound height was 5.4 ± 0.7 cm.

Nests were centered under nest entrances and had an average depth of 23.0 ± 1.6 cm (N = 19 nests). The upper portion of the nest (the first 4 to 5 cm below ground) was a collection of small chambers, side by side, that were often supported by many fine roots. These chambers were between 1 and 2 cm deep and collectively filled an area from 5 to 10 cm in diameter.

A number of distinctive chambers were located below this area and were found at depths ranging from 8 – 43 cm. The sides of these chambers had an average width of 4.3 ± 0.6 cm, an average height of 1.6 ± 0.2 cm, and were roughly ovoid in shape. These were connected to the central shaft at one side of their longest axis, but were not directly connected to any other chambers. Each chamber was offset in a vertical plane from any chambers that were directly above or below.

## Discussion

For myrmecochorous seed dispersal to be successful for a plant, elaiosome-bearing seeds must be picked up by ants, carried to the nest, and then discarded somewhere where germination is possible or even favored. Each of these steps is influenced by the biology of the seed-dispersing ants, characteristics that in nearly every case remain enigmatic or simply unstudied. Here biological features of an ant that has the potential to disperse the seeds of thousands of plant species in western Australia are documented. In the following sections particular aspects of the biology *of R. violacea* and how each of these characteristics can influence the fate of myrmecochorous seeds are discussed.

## Foraging

The first step in dispersal is the removal of seeds by the ants (which is a function of the foraging behavior of the ants), the spatial distribution of nests, and the number of workers from a colony that forage. The results of this study suggest that *R. violacea* do not specifically search for seeds. Workers instead scavenge for any available food in a process that will sometimes, but only seasonally, yield seeds. During the foraging component of the study (just before seed maturation, as a result of a late seed set in 2007), the researchers found that insects and insect parts were the most common items retrieved by foragers. If the survey had been repeated later in the season, more seeds being retrieved by *R. violacea* (Gove et al. 2007) would undoubtedly have been found. Nonetheless, the point remains that much of *R. violacea* foraging, perhaps throughout most of the year, is for items other than seeds. What was regarded as collection ‘errors’, where foragers collected plant parts and small clumps of sand (Figure 2), accounted for a quarter of all seemingly successful foraging trips. *R. violacea* is either not careful in discriminating between food and non-food items or it may also be foraging for resources that are used for other purposes besides food. We suspect the former, as a particular use (e.g., nest structures) for non-food material during nest excavations could not be identified.

Our past and present results suggest *R. violacea*, although a keystone seed disperser from the plant's perspective, is not an obligate elaiosome specialist. *R. violacea* is a generalist forager that makes quick rather than careful choices as to what it picks up, and then hurriedly brings back to the nest. Such foraging is a scramble, rather than an interference, competition strategy, which fits well with the propensity of *R. violacea* to avoid interspecific encounters with other ants (D Lubertazzi, personal observation). Since it is relatively cheap to forage (Nielsen 1997), but potentially dangerous to fight over food, such an approach may be successful for behaviorally subordinate ants like *R. violacea*.

## Re-dispersal

In a generalized model of myrmecochory (e.g., Beattie and Culver 1982), seeds may be deposited by ants in nutrient-enriched garbage piles inside their nests. In practice, seeds can also be disposed of by workers removing them from the nest (re-dispersed). We found that *R. violacea* regularly move seed-like refuse outside their nest with some beads being discarded more than 2 m from the nest entrance. Seeds brought into the nest, once stripped of their elaiosome, may be discarded just as the beads were (often seeds are discarded within 12 hours of being collected, A Gove, personal observation). Secondary redispersal can be influenced by the size and shape of a diaspore after its elaiosome has been removed. Round and smooth diaspores, for example, may remain buried at higher rates than those that contain surfaces and structures that the ants can easily grasp with their mandibles (Gomez et al. 2005).

Assuming re-dispersal is random in its direction relative to the initial dispersal event, it may increase total seed-dispersal distance, but reduce the probability that seeds enjoy the benefits that come from being deposited within the nest. Yet there can also be benefits available to being ejected from the nest. Some re-dispersed beads became buried in the mound of nest spoil, and any seeds suffering this fate may still avoid mortality from fire and/or the harsh conditions during the long inter-fire interval. Removed seeds are also not all aggregated within the nest, which potentially lessens competition between germinating seedlings.

## Nest demography

Two studies have recently found that activity and abundance of *Rhytidoponera* foragers in general (Gove et al. 2007), and *R. violacea* in particular (McCoy 2008), are the best predictors of the rate of seed dispersal in this study region and more generally. The abundance of *Rhytidoponera* foragers is a function of colony densities and nest demography. *R. violacea* colonies were small (mean of 190 workers) and foragers had a relatively small foraging range, such that high densities of *R. violacea* can only occur where colonies are dense. One consequence of the small nest size is that seeds from even a single plant may be distributed to multiple nests (Gove et al. 2007; RR Dunn, personal observation). This stands in contrast to the fate of seeds collected by Australian meat ants (*Iridomyrmex purpureus* species group), which have a large foraging range and colony size. These seed-collecting *Iridomyrmex* species are likely to concentrate seeds in and around a single nest that is located in the middle of a large foraging area (Whitney 2002). Colony size, with its implications for nesting biology and foraging dynamics, can play a key role in how ants provide mutualism benefits to their partners. Plant distribution patterns, seedling competition, and gene-flow can all be influenced by species-specific patterns of ant dispersal and re-dispersal of seeds.

## Nest architecture

The architecture of an ant nest can influence where dispersed seeds are placed in the soil profile (at least those seeds which are not redispersed), which, in turn, affects the probability that a seed may germinate in the next fire or persist in the soil through several fire intervals (Figure 4). Nests of *R. violacea* are relatively shallow and typically have a mound with a single entrance. Nest construction appears to follow a simple template; a collection of small interconnected chambers just under the ground surface, a single main shaft leading down from the central ground entrance, and a series of three to five progressively deeper chambers. While it is possible to build deeper (e.g., > 1m in depth for *Melophorus* spp.) and more long lasting nests in the sandplains, *R. violacea* seems to favor a nest-building strategy that limits extensive construction and maintenance. Our observations of nest migrations in the field, and other studies documenting nest movements by *Rhytidoponera* (Searle 1978; Ward 1981b; Thomas 2002) also suggest that they can, and will, readily move their nests to a new location. If colonies periodically move to a new nest then the seeds they disperse can be buried in a wider range of locations. This could, like having many small colonies, reduce plant sibling-competition and increase gene-flow.

Chambers at and near the surface of nests of *R. violacea* are well positioned for the germination of seeds. Fire cues for germination can penetrate at least as deep as 12 cm in hot fires (McCoy 2008), but germination cues are likely to vary in their depth between fires and between patches within fires. Germination is likely to be optimized for plants when seeds are buried at a range of depths (as occurs in *R. violacea* nests) such that in any given fire at least some seeds will germinate (McCoy 2008). Movement of nests by *R. violacea* may yield similar effects to those that result from burial at a variety of depths. Seeds from a single plant scattered among patches of soil are likely to have different fates with regard to fire timing and intensity. Where *R. violacea* is present, a single myrmecochorous plant may have seeds dispersed to different underground depths and locations over the course of a number of years, with the consequence that even an extremely hot fire will not kill all seeds and even a relatively cool fire will trigger the germination of some seeds.

Seeds that are at the bottom of nests may remain dormant across many fires, since the longevity of fire-adapted seeds can be tens and even hundreds of years (McCoy 2008). The occasional deep burial of seeds (Figure 4) increases the probability that for any cohort of seeds, some individuals disperse across several fires and hence through time. Such dispersal through time may reduce the probability of local extinction of ant-dispersed lineages. Lower local extinction rates for antdispersed plants than for plants dispersed by other means might account, in part, for two surprising findings for ant-dispersed plants. First, Gove et al. (2009) found that antdispersed plant species do not necessarily have smaller geographic ranges than related species with other dispersal modes, despite short average dispersal distances. Second, Lengyel et al. (2009) found that ant-dispersed plant lineages have more rapid net diversification rates (speciation or extinction) than do lineages with other dispersal modes. Both of these patterns could be explained if ant-dispersed plants have reduced local extinction rates relative to other plants.

## Conclusion

*R. violacea* possess numerous traits that complement their role as seed dispersers for many plant species. Foragers are omnivorous and favor loose discrimination in determining what is, or is not, food. When they find a potential food item (e.g. an eliaosome-bearing seed) they quickly bring it back to their nest. It can also be beneficial for plant fitness that *R. violacea* nests are relatively shallow and this species possesses life history attributes of an r-strategy species (i.e., living in small colonies (low biomass), maintaining relatively ephemeral nests (short nest “life”), and occurring in high nesting densities (weed-like populations)). With these traits, foragers can be available to interact with a plant's diaspores across a large area, and the seeds they transport may be carried to and buried in many favorable locations.

While ant assemblages often include many omnivorous scavenging ant species that cooccur, such ants can differ greatly in their biology and hence how they affect any seeds they collect. *Aphaenogaster rudis*, a keystone seed disperser in eastern North America, shares many traits with *R. violacea* including small and shallow nests, occasionally high local densities, and rapid and relatively indiscriminate discovery of food (Zelikova et al. 2008, Ness et al. in press). Whether the seed dispersing ants in other regions where myrmecochory is common, such as the temperate forests of Asia, Europe, and the Fynbos of South Africa, possess similar traits deserves further study.
